# Editorial: Non-specific symptoms of unruptured intracranial aneurysms-new concepts in pathophysiology, hemodynamics and potential warning signs

**DOI:** 10.3389/fsurg.2025.1572304

**Published:** 2025-03-17

**Authors:** Grigorios Gkasdaris, Chloe Dumot, Ntenis Nerntengian, Theodossios Birbilis

**Affiliations:** ^1^ Department of Neurosurgery, Democritus University of Thrace, University Hospital of Alexandroupolis, Alexandroupolis, Greece; ^2^ Department of Neurosurgery, Pierre Wertheimer Neurological and Neurosurgical Hospital, Hospices Civils de Lyon, Lyon, France; ^3^ Department of Neurosurgery, University of Marburg, Marburg, Germany

**Keywords:** intracranial aneurysms, cerebral aneurysms, unruptured, symptoms, non-specific, atypical headaches, vertigo

**Editorial on the Research Topic**
Non-specific symptoms of unruptured intracranial aneurysms

This Research Topic aimed to shed light on the symptoms or warning signs, the pathophysiology, and the hemodynamics of the unruptured intracranial aneurysms, with particular interest in their non-specific symptomatology.

Unruptured intracranial aneurysms are usually asymptomatic, however certain symptoms and signs may occur including visual dysfunction, oculomotor nerve palsy, epilepsy, cerebral infarcts, transient ischemia and unusual headaches (1, 2). Some of the relevant symptomatology often remains ambiguous. Headache is maybe the most common presenting symptom, but direct causality is not clear (3, 4). Vertigo has also been reported as a potentially associated symptom (5–8). For example, Lu et al. presented a very rare case of a 54-year-old woman with an unruptured aneurysm of the anterior inferior cerebellar artery (AICA) following stereotactic radiosurgery treatment for a vestibular schwannoma, who presented sudden onset of severe vertigo and vomiting, accompanied by unsteady gait. Considering the clinical presentation and the tumor volume, microsurgical resection was chosen. During debulking, a dissecting “fusiform-like” aneurysm of the AICA was encountered accidentally within the tumor and was successfully treated using a clip. After the surgery, the preoperative symptoms of vertigo, vomiting and unsteady gait never recurred after surgery. The cause of unruptured intracranial aneurysm symptoms is often attributed to local mechanical phenomena or even hemodynamic alterations.

Hacket et al., from the Barrow Neurological Institute (Phoenix, US), provided insight on those atypical symptoms. They conducted a retrospective review based on 454 patients with unruptured intracranial aneurysms who underwent microsurgical treatment, with emphasis on the prevalence of non-specific symptoms. From the initial pool of patients, 350 (77%) were symptomatic, having as the two most common symptoms headache [211 (46%)], and vertigo [94 (21%)], followed by cognitive disturbance [68 (15%)], and visual disturbance [64 (14%)]. It is unclear whether symptoms were a direct result of the aneurysms, nevertheless the analysis showed that 258 patients (79%) experienced symptom resolution or improvement during the follow-up.

To better understand this ambiguous symptomatology, knowledge of the relevant physiopathology is required. In clinical practice, it is difficult to determine the rupture risk of an aneurysm. This is of paramount importance in deciding between treatment and surveillance. In cases of unclear symptom presentation, further rupture risk assessment or hemodynamic understanding could perhaps help to the decision-making, medical care and the depiction of a possible connection between these symptoms and unruptured intracranial aneurysms.

Concerning aneurysm pathophysiology and formation, Cannizzaro et al., in a group of 22 patients diagnosed with ruptured (*n* = 12) and unruptured (*n* = 10) intracranial aneurysms who had undergone microsurgical clipping, evaluated pre-operative MRI wall aneurysmal enhancement (T1-weighted with gadolinium), and CD68 positivity. This observational study showed a correlation of CD-68 positivity and wall enhancement with aneurysm growth and rupture, emphasizing the influential role of inflammation in aneurysmal genesis and progression.

Guo et al. performed an analysis of computational fluid dynamics in angiographic data of 80 patients harboring bifurcation aneurysms and 80 control subjects with no aneurysms. They depicted that pressure was the highest at the center of direct flow impingement on the bifurcation wall, and dropped rapidly toward the branches. Furthermore, it was shown that bifurcation angle was significantly enlarged in patients with bifurcation aneurysms than those without, and most aneurysms leaned toward the smaller arterial branch or the arterial branch that formed a smaller angle with the parent artery, where the hemodynamic stresses increased significantly.

In an effort to predict aneurysmal rupture risk, several papers in this research topic are presented.

Fan et al. performed an analysis of the wall thickness of intracranial aneurysms using computational fluid dynamics simulation, in order to explore the relationship between the simulated thickness of the aneurysm wall, the translucent area, and the rupture point of the real aneurysm's surface. Using 48 aneurysms, 20 were ruptured and 28 unruptured ones treated surgically, they concluded that the simulated thickness could be used as an effective tool to predict the rupture point or detect a translucent area of an aneurysm's surface that could rupture in the future.

Then, Shen et al. studied a total of 649 patients with 964 intracranial aneurysms (ruptured and unruptured) using a novel parameter named MAPN ratio (the ratio of mean arterial pressure to neck width). They showed that MAPN was higher in the ruptured aneurysm group. Additionally, they constructed a prediction model including as parameters the aneurysm size and location, the presence of secondary sacs and the MAPN. Interestingly, this model exhibited superior performance compared to the UCAS and the PHASES score. In general, they suggested the further use of MAPN as a new tool for evaluating the rupture risk.

Finally, Chen et al. studied the dynamics of the aneurysm wall by estimating the irregular pulsation during the cardiac cycle as a potential predictor of aneurysm rupture. Amongst 11 unruptured aneurysms, 5 aneurysms (45.45%) showed irregular pulsation. No significant differences in morpho-hemodynamic characteristics were observed between aneurysms with or without irregular pulsation. Nevertheless, more remarkable changes in aneurysm size, volume, oscillatory shear index and rupture resemblance, over the cardiac cycle, were significantly linked to irregular pulsation, indicating that the latter may indicate hemodynamic instability within the aneurysm.

To conclude, the aforementioned studies contribute to the further understanding of the field of unruptured intracranial aneurysm pathophysiology and symptomatology, nevertheless the diversity of symptoms, the uncertainty regarding direct causality and the complexity of hemodynamics should encourage future studies for more investigation.
